# N2 modified cap analogues as translation inhibitors and substrates for preparation of therapeutic mRNA

**DOI:** 10.1007/s00249-023-01676-7

**Published:** 2023-09-01

**Authors:** Karol Kurpiejewski, Marzena Jankowska-Anyszka, Renata Grzela

**Affiliations:** 1 Faculty of Chemistry, University of Warsaw, 02-093 Warsaw, Poland; 2 Division of Biophysics, Institute of Experimental Physics, University of Warsaw, 02-093 Warsaw, Poland

**Keywords:** mRNA, Cap analogues, Gene therapy, Inhibitors, mRNA vaccines, mRNA engineering

## Abstract

In recent years many scientists have begun to focus on the mRNA molecule’s emeregence as a new type of drug. Its fast-moving and successful career as a vaccine technology cannot be underestimated. mRNA provides new opportunities and allows for the rapid preparation of effective drugs at low cost. These extensive possibilities stem from a number of factors, but the small cap structure located at the 5′ end of the mRNA is one contributing factor. Cap protects mRNA and ensures efficient recruitment to the biosynthesis machinery. Furthermore, it allows for the easy introduction of various modifications that influence the activity of the entire mRNA. Among the many different cap analogues that have been reported, those modified at the N2 position of guanosine have been systematically developed. N2-modified caps in the form of nucleoside monophosphates or dinucleotides show favorable biological properties, as well as a high capacity to inhibit the translation process in the cell-free RRL system. Modified N2 dinucleotides are efficiently incorporated into the structure of the mRNA transcript, and in specific circumstances with the correct orientation, making them an interesting alternative for ARCA-type analogues. Moreover, mRNA transcripts containing cap structures modified within the exocyclic amino group show very high translational activity. Therefore, analogues modified at the N2 position may have future applications as therapeutics against various manifestations of cancer and as desirable tools in RNA engineering.

## Introduction

mRNA-based technologies have undergone intense research around the world for nearly three decades. During the outbreak of the COVID-19 pandemic, this technology experienced overwhelming development and implementation, which resulted in a highly effective vaccine that saved countless lives on a global scale (Baden et al. 2021; Polack et al. 2020). The overall process was extremely dynamic, in which mRNA as a therapeutic agent was first proposed in 1989 and has been widely used for in vitro transfection techniques, and after three decades, mRNA was finally classified as a drug (Schlake et al. 2012).

Compared to their DNA counterparts, mRNA-based vaccines exhibit many advantages, making them effective therapeutics. The main disadvantages of DNA-based vaccines are that they pose severe risks, such as toxicity at high doses, and they can integrate into the host genome, which can lead to the activation of oncogenes (Raper et al. 2003). Fortunately, mRNA-based vaccines do not pose such a threat (Sahin et al. 2014; Weissman 2015). In addition, the delivery of these therapeutics is easier because mRNA translates immediately upon reaching the cytosol, without the need for nuclear transcription and subsequent export from the nucleus to the cytoplasm. In addition, mRNA is produced relatively easily via an in vitro transcription (IVT) reaction. This process, as well as the mRNA purification procedure, can be optimized independently of the encoded antigens. Moreover, mRNA is easily modified, which affects its various properties, such as translation efficiency and stability. This advantage is not available for DNA vectors. Hence, it is possible to optimize the mRNA molecule for specific applications.

**Fig. 1:**
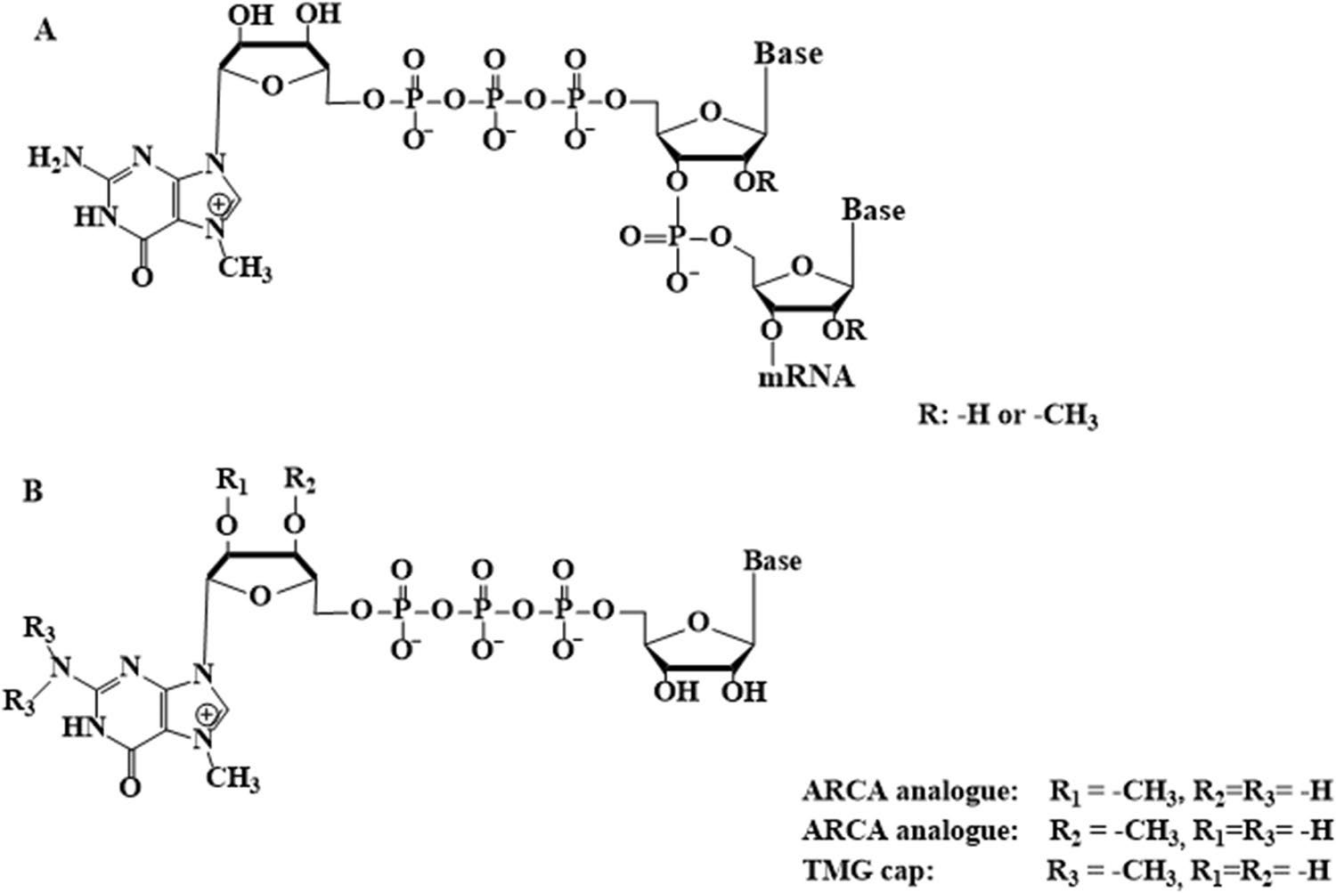
Cap structures. **A** Structures of cap at 5′ end of mRNA, **B** Structures of cap analogues: ARCA and TMG cap

**Fig. 2:**
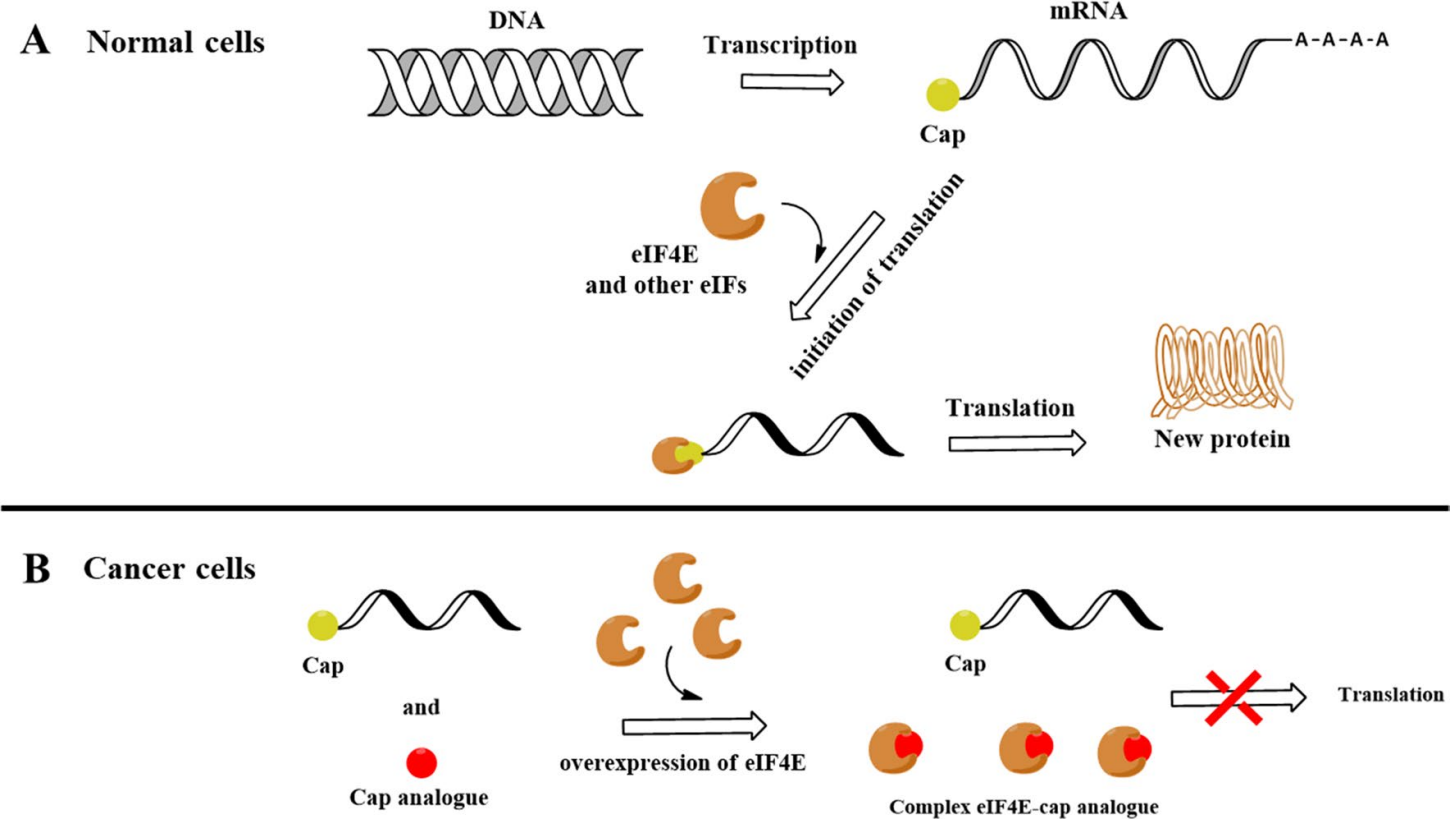
**A** Mechanism of cap-mediated gene expression in a healthy cell. **B** Cap analogues with improved properties compared to normal cap-mRNA in a cancer cell

The 5’ end, also known as cap (Fig. 1A), of mRNA structure is essential for maintaining functionality and enabling improved properties. The cap structure consists of a 7-methylguanosine linked by 5′-5′ triphosphate bridge to the first transcribed nucleoside; this reverse orientation linkage between the cap and the transcript plays a key role in the mRNA’s stability (Furuichi 2015). Additionally, the cap is recognized by highly specialized proteins that bind and allow it to participate in many processes, including maturation, nuclear export, translation initiation, and mRNA protection (Galloway and Cowling 2019). According to reports, cap possesses a variety of functions involving every phase of mRNA metabolism, but in the case of RNA-based vaccines, the most important functions are those that enhance translation initiation and protect against 5′-to-3′ exonucleolytic degradation. Over the years, numerous researchers harnessed these functions through the introduction of modifications. For example, translation initiation is enhanced by cap modification, promoting a higher affinity for eukaryotic translation initiation factor 4E (eIF4E) (Shanmugasundaram et al. 2022) (Fig. 2A).

The most common method for the preparation of capped mRNA is the incorporation of a dinucleotide (e.g., m^7^GpppG/A) by RNA polymerase (e.g., T3, T7, or SP6) during IVT reaction. As a result, functional mRNAs are generated with a 5′ end properly integrated with the one that is incorporated in the reverse orientation. Anti-reverse cap analogues (ARCA) possessing a O-methyl group at the C2′ or C3′ position of 7-methylguanosine (Fig. 1B) were the first dinucleotide cap analogues that allowed incorporation in proper orientation during IVT (Grudzien-Nogalska et al. 2007). Later studies revealed that this modification modulates the cap interaction network with various proteins and thus positively affects the overall level of translation (Miedziak et al. 2020). Not surprisingly, the 3′-O-methylated m^7^G-ribose was used as a cap analogue in current mRNA vaccines against COVID-19 (Sahin et al. 2020).

Over the past two decades, various modifications of the cap structure have been developed and tested, displaying beneficial properties. In recent years, studies have shown that modifications introduced within an exocyclic amine group of m^7^G lead to enhanced translational inhibition based on the increased affinity of the modified cap structure with the eukaryotic translation initiating factor 4E. This can be used to block elevated levels of this protein, which contributes to oncogenesis (Fig. 2) (Lazaris-Karatzas et al. 1990). In addition, modifications at the N2 position of 7-methylguanosine contribute to the efficiency of the translation process because it is efficiently incorporated into the mRNA chain and predominantly in the correct orientation.

This brief review presents properties and potential applications of new cap analogues modified at the N2 position of 7-methylguanosine.

## N2-modified monophosphate cap analogues

Research on the effects of substitutions at the N2 position of 7-methylguanosine has been conducted for more than a decade. Such types of analogues were initially obtained in connection with studies of nematodes, in which more than 70% of mRNAs have an unusual hypermethylated version of the cap structure (Fig. 1B) containing 7, N^2^, N^2^-trimethylguanosine (TMG or m_3_^2,2,7^G). The effect of TMG cap on the eIF4E protein in parasitic nematode *Ascaris suum* was intensively studied (Liu et al. 2011). *Ascaris* cap-binding proteins must contend with two distinct populations of mRNAs (containing m^7^G (MMG) and m_3_^2,2,7^G caps), suggesting that these proteins are unique as eIF4E of higher eukaryotes does not bind efficiently TMG-capped mRNA. Due to the presence of TMG cap structures in parasitic nematodes, which pose a serious health and economic problem, the search for effective translation inhibitors began by focusing on the N2 position differentiating m^7^G- and m_3_^2,2,7^G-cap.

**Fig. 3:**
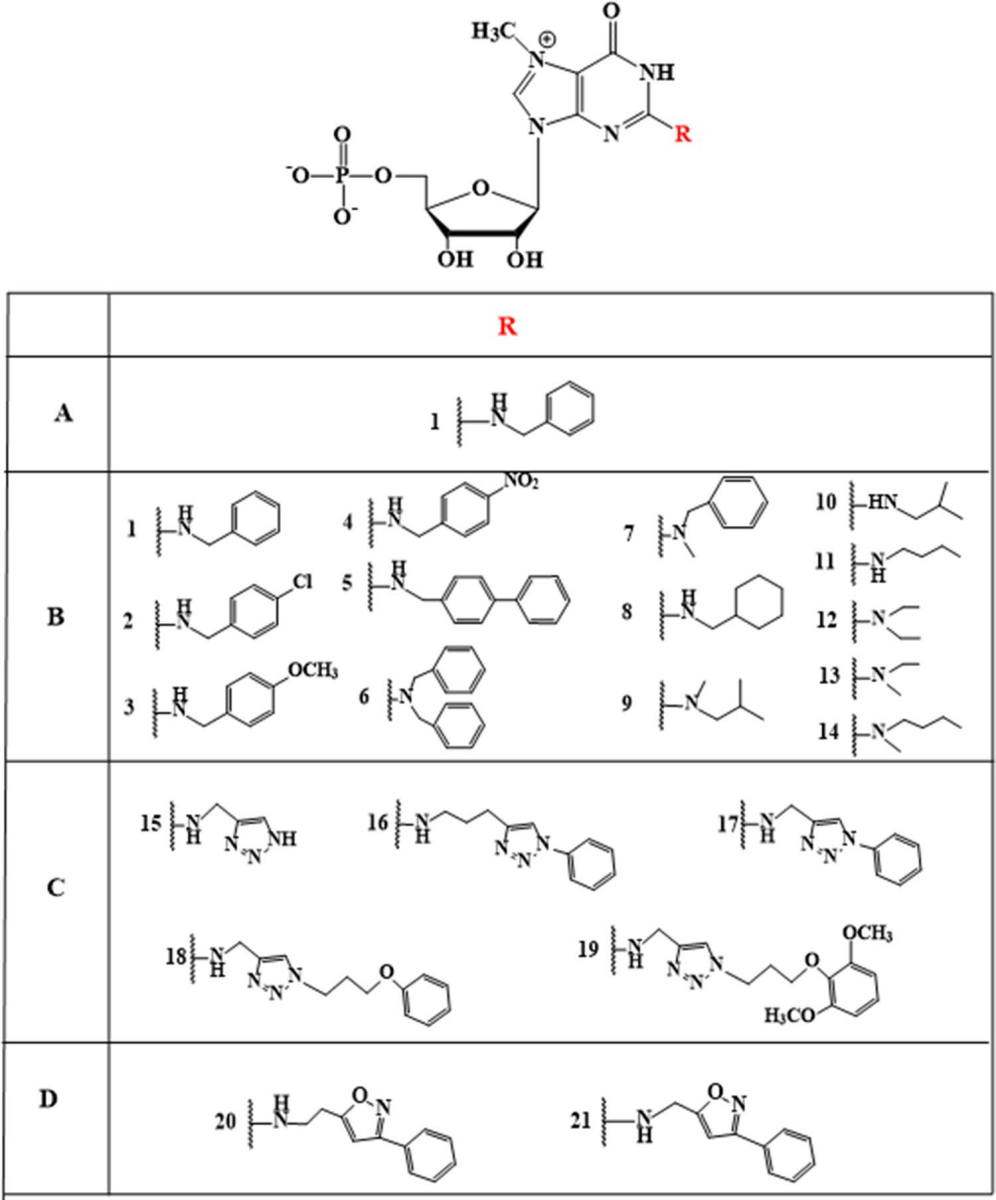
Mononucleotide cap analogues modified at N2 position of 7-methylguanosine

During studies of the intermolecular interaction of MMG/TMG caps with *Ascaris* eIF4E, it was found that the addition of an N2-benzyl substituent (1) to the nucleoside monophosphate (Fig. 3 row A) led to significant inhibition of the translation in the *Ascaris suum* embryo cell-free system. In 2012, fourteen N(2)-modified mononucleotide analogues with various alkyl, cyclic, and aromatic substituents at the N2 position (Fig. 3 row B) were prepared and tested in the same system (Piecyk et al. 2012). The results showed that the analogues bearing aromatic or larger aliphatic substituents (Table 1) effectively inhibited the translation process, in which the most effective compound with IC_50_∼0.9 μM was N(2)-*p*-methoxybenzyl-7-methylguanosine-5′-monophosphate (3) (IC_50_ of m^7^GTP that is very effective inhibitor was ~ 6.1 μM). In addition, it was noted (as previously postulated by Cai et al. 1999) that the addition of a second substituent at the N2 position of guanosine significantly reduced the level of inhibition due to the loss of one hydrogen bond between the guanine amino group and the E116 carboxyl group in eIF4E protein. The results laid the groundwork for further development of cap analogues having only one phosphate (according to many previous reports, nucleoside monophosphates usually show only modest inhibitory activity) and aromatic substituents at the N2 position for potential use for inhibition of translation in mammalian cells.

**Table 1:** Inhibitory properties of N2-substituted m^7^GMP analogues

Compound	IC_50_ [µM]	
	Ascaris suum	Rabbit reticulocyte lysate (RRL)
m^7^GMP	> 50	> 50/14.2 ± 2.0^DMF^
m^7^GTP	6.1	2.3 ± 0.2
1	2.9	−
2	3.8	−
3	0.9	−
4	12.6	−
5	4.3	−
6	> 50	−
7	> 50	−
8	5.7	−
9	>50	−
10	4.5	−
11	3.9	−
12	> 50	−
13	13.9	−
14	> 50	−
15	−	14.4 ± 1.1
16	−	8.5 ± 1.6
17	−	10.5 ± 1.3
18	−	5.0 ± 0.5
19	−	2.0 ± 0.1
20	−	3.7 ± 0.1^DMF^
21	−	1.9 ± 0.1^DMF^
m^7^GpppG	−	8.94 ± 0.86/2.70 ± 0.1^DMF^

In 2014, Piecyk et al. reported the synthesis of new triazole ring-based (Fig. 3 row C) N2-modified 7-methyl guanosine monophosphates (Piecyk et al. 2014). The triazole rings showed excellent biological properties in the context of medicinal chemistry (Hein & Fokin 2010; Tron et al. 2008). The authors described five new triazole-containing nucleoside monophosphates cap analogues that were prepared using “click chemistry”. Their ability to inhibit cap-dependent translation was evaluated in rabbit reticulocyte lysate (RRL) (Kowalska et al. 2009). Obtained results showed that the translational inhibitory potency were similar to that of m^7^GTP. Based on these results, it was concluded that aromatic substituents at the N2 position of guanine compensated for the lack of two phosphate groups and effectively inhibited protein synthesis.

In 2020, further analogues containing isoxazole rings at the N2 position (Fig. 3 row D) were described (Piecyk et al. 2020). In general, isoxazole derivatives exhibit numerous biological activities and act as antimicrobial, antiviral, anticancer, immunomodulatory, and antidiabetic agents (Zhu et al. 2018; Zimecki et al. 2018). The study described the preparation of two new nucleoside monophosphate analogues with isoxazole as well as their biological evaluation as inhibitors of protein synthesis in the RRL system and thermal stabilization of eIF4E by differential scanning fluorimetry (DSF). Usually, such experiments are carried out using aqueous solutions of cap analogues, but in this case, the obtained compounds were only partially soluble in water and required a small amount of dimethylformamide (DMF) at a concentration of 0.85%. The prepared compounds were effective inhibitors, with determined IC_50_ values similar to those for m^7^GpppG. Importantly, the analogues differed only in the number of carbon atoms in the linker between the purine base and the isoxazole substituent, and the compound with the slightly longer linker showed a twofold inhibition compared to the shorter one. Given the reduced solubility of the isoxazole-containing analogues, they appeared to be considered slightly inferior inhibitors in the indirect comparison to triazole-containing compounds, even though they displayed better inhibitory properties despite being nucleoside monophosphates. Additionally, the analogues were tested for thermal stabilization of the eIF4E protein using the DSF method. The results indicated that both mononucleotide compounds specifically bind to the translation initiation factor eIF4E, to a similar extent as the extensively studied dinucleotide m^7^GpppG.

## N2-modified dinucleotides cap analogues

N2-modified monophosphate analogues of cap containing aromatic substituents can effectively inhibit the translation process in the cell-free RRL system in vitro. Hence, this attribute was exploited to produce dinucleotide analogues of cap to prepare mRNA transcripts with high translational activity.

**Fig. 4:**
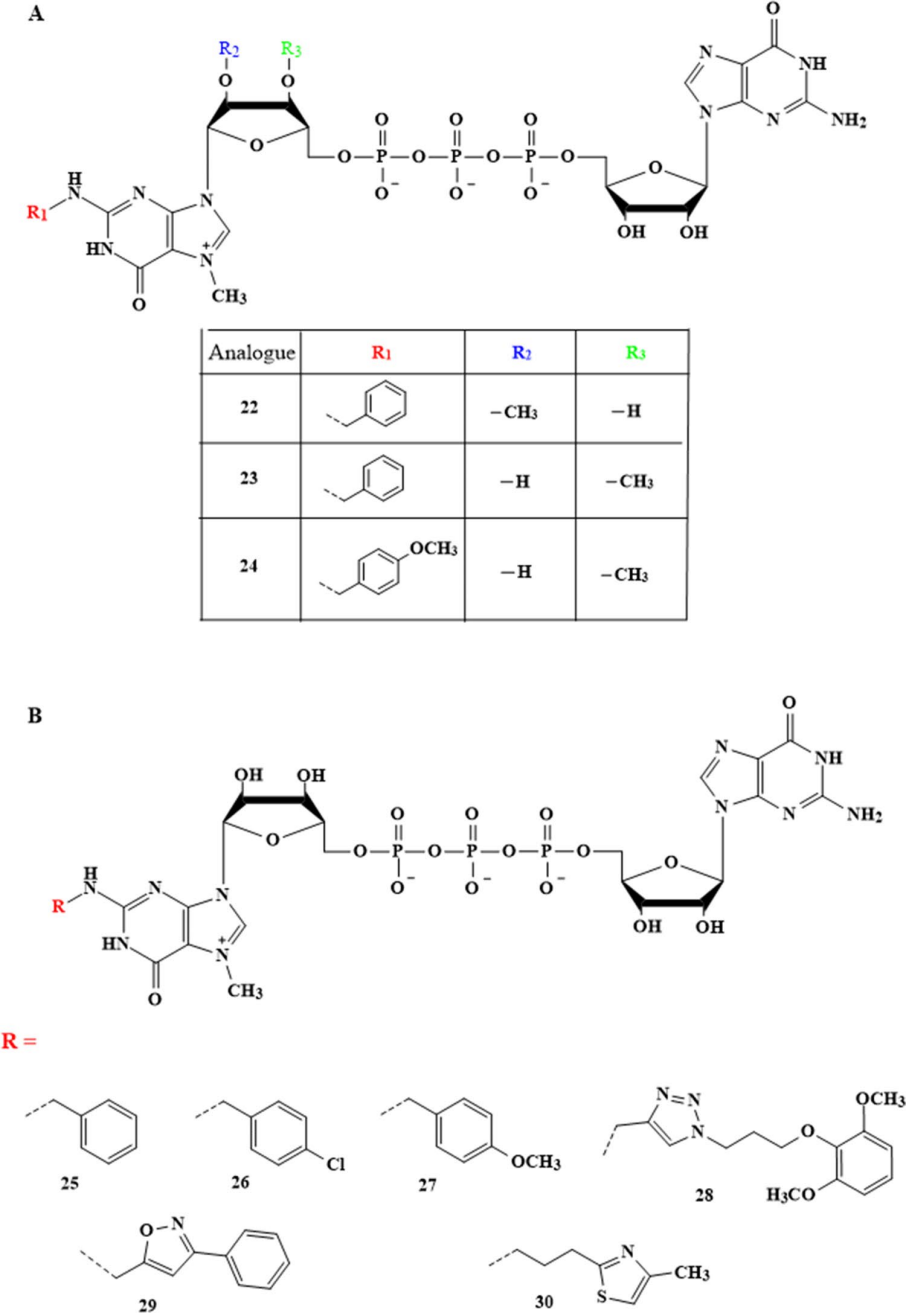
Dinucleotide cap analogues modified at N2 position of 7-methylguanosine

The first reported case of the aforementioned 5′-end mRNA analogues, modification at the N2 position, was published in 2018 (Fig. 4A). Kocmik et al. described dinucleotides that contained two types of modifications within m^7^G: 3′- or 2′-O -methylation and, in addition, substitution at the N2 position (benzyl or p-methoxybenzyl) (Kocmik et al. 2018). The modification within the ribose provided 100% correct incorporation orientation, while the modification within N2 resulted in greater affinity for the translation initiation factor. During their research, the authors obtained, by chemical synthesis, three new dinucleotide analogues of cap, i.e., bn^2^m_2_^7,2ʹO^GpppG (22) bn^2^m_2_^7,3ʹO^GpppG (23), and (p-OCH3bn)^2^m_2_^7,3ʹO^GpppG (24). These dinucleotides were tested in vitro for translation inhibition in the RRL system.

The results showed that compared to the standard (m^7^GpppG), the obtained compounds exhibited a 2.5-fold increase in inhibitory properties, while for bn^2^m_2_^7,2ʹO^GpppG (22) analogue it was 6.8-fold (Table 2). The authors also tested the influence of the obtained compounds on the translation properties of analogue-capped mRNAs. The experiments were conducted in two systems, the cell-free RRL and the cellular HEK293 environment. ARCA-capped mRNAs were used as the control. The data from RRL system revealed a slightly higher level of translation relative to the standard for mRNA modified at the 5′ end of bn^2^m_2_^7,2ʹO^GpppG (22), while both transcripts containing bn^2^m_2_^7,3ʹO^GpppG (23) and (p-OCH3bn)^2^m_2_^7,3ʹO^GpppG (24) showed slightly lower translation efficiency (Table 2). This stemmed from the translation efficiency of the ability of the dinucleotide analogues to incorporate into the mRNA structure. In the case of bn^2^m_2_^7,2ʹO^GpppG (22), which was very well accepted by RNA polymerase, the estimated efficiency of incorporation of this dinucleotide into the RNA chain was almost 100% efficiency, while that of the other dinucleotides was approx. 50%. In another test, the efficiency of translation in the cellular environment was examined using HEK293 lines. As a result, all the newly obtained mRNA transcripts having the tested dinucleotide analogues of cap at the 5' end showed higher translation efficiency compared to the standard cap m^7^GpppG (Table 2).

**Table 2:** Inhibitory and translation properties of dinucleotide cap analogues modified at N2 position of 7-methylguanosine

Compound	IC_50_ [µM]		Translational properties of mRNA capped		
	Ascaris suum	Rabbit reticulocyte lysate (RRL)	RRL		HEK293 cells
			Relative translation efficiency	Relative cap-dependent translation efficiency	Relative total protein expression
m^7^GMP	>50	>50	−	−	−
m^7^GTP	6.1	2.3 ± 0.20	−	−	−
m^7^GpppG	−	8.94 ± 0.86	1	1	1
ARCA	−	−	1.56 ± 0.03	1.65 ± 0.06	1.46 ± 0.50
22	−	1.7 ± 0.16	1.72 ± 0.28	1.82 ± 0.34	2.87 ± 0.50
23	−	4.46 ± 0.53	1.39 ± 0.19	1.44 ± 0.22	3.30 ± 0.86
24	−	4.12 ± 0.35	1.29 ± 0.17	1.35 ± 0.20	2.42 ± 0.30
25	−	0.61 ± 0,04	3.30 ± 0.77	3.52 ± 0.82	1.80 ± 0.34
26	−	0.89 ± 0,12	2.76 ± 0.54	2.94 ± 0.53	2.18 ± 0.91
27	−	0.98 ± 0,13	3.29 ± 0.74	3.52 ± 0.73	2.29 ± 0.12
28	−	1.30 ± 0,23	2.65 ± 0.89	2.80 ± 0.90	1.96 ± 0.05
29	−	0.57 ± 0,06	3.37 ± 0.84	3.51 ± 0.82	1.64 ± 0.09
30	−	1.87 ± 0,08	1.92 ± 0.43	2.00 ± 0.44	1.42 ± 0.20

The stability of the studied compounds was also examined. It is known that overall protein synthesis depends, among other aspects, on the half-life of the protein-coding transcript, hence, the studied compounds were subjected to enzymatic hydrolysis in vitro using Dcp1/Dcp2 decapping complex. The results showed that the introduction of the modification at the N2 position of guanine decreased the protection of the structure against the decapping complex compared to the standard cap. In contrast, the introduced modifications enhanced stability of the capped transcripts in HEK293 cells, which become higher compared to that of the transcripts capped with regular cap or with ARCA. This was probably the result of increased affinity of analogue-capped mRNA to eIF4E and its protective character for decapping.

**Fig. 5:**
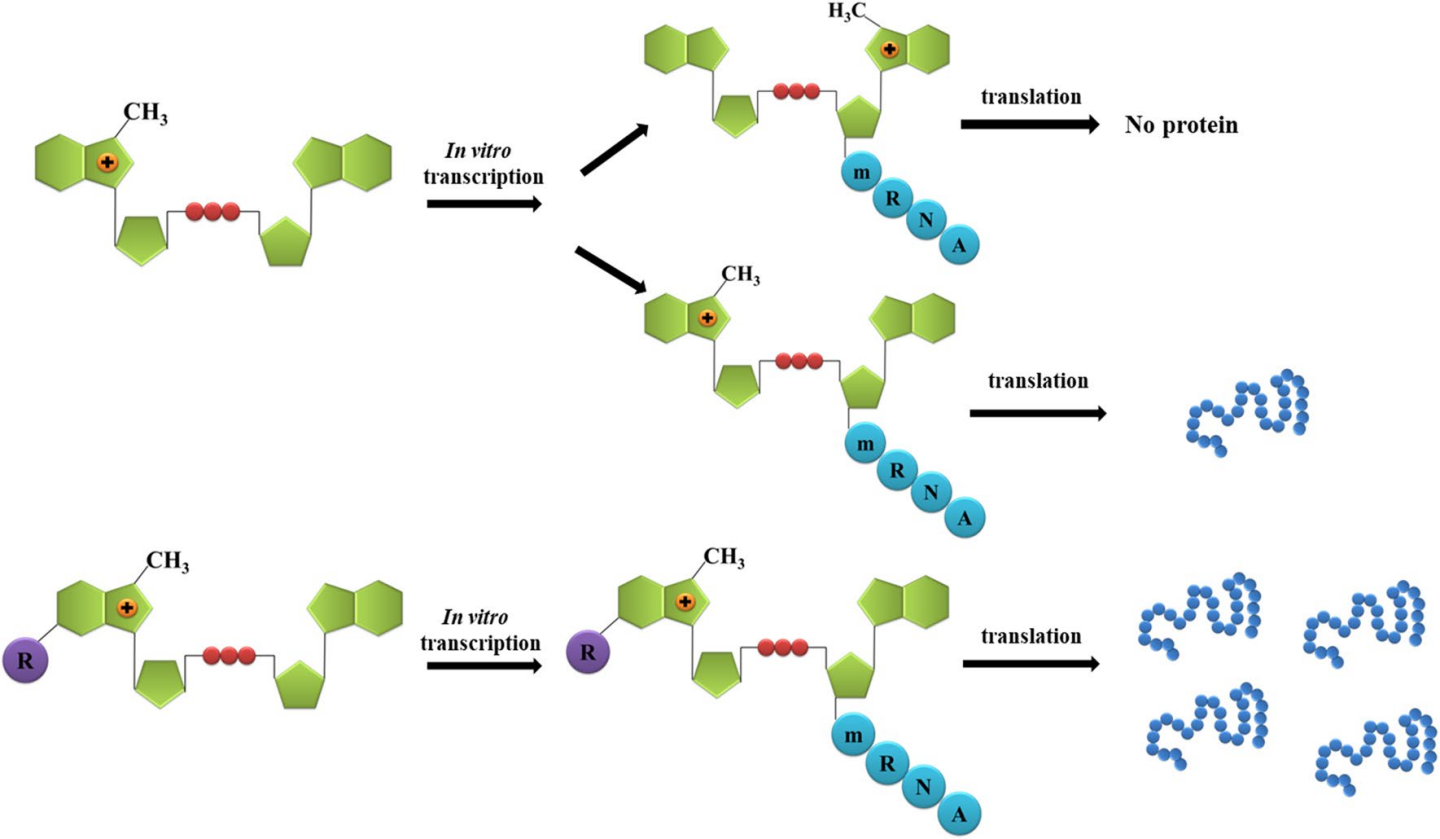
Effect of N2-modified dinucleotide analogues of cap compared to m^7^GpppG

In 2022, a broader set of six cap analogues modified only on the exocyclic amine group was synthesised by Jankowska-Anyszka’s group (Grzela et al. 2022). The examined dinucleotide analogues contained substituents that ranged from benzyl (methoxy-, chloro-) derivatives to those with more extended side chains, such as the appropriately substituted triazole, isoxazole, or thiazole (Fig. 4B). The obtained compounds were tested for their inhibitory properties, thermostabilization of eIF4E, translational properties, and incorporation into the mRNA transcript. The abilities of analogues to inhibit translation were studied in rabbit reticulocyte lysate and compared to the standard cap m^7^GpppG. It was found that all dinucleotides inhibited translation more efficiently than m^7^GpppG (Table 2). In the case of dinucleotides modified with an isoxazole (29) or a benzyl ring (25), the inhibitory properties were nearly 15-fold higher (IC_50_ of 0.57 and 0.61 µM, respectively) compared to the dinucleotide unmodified at the N2 position. Slightly weaker translational inhibitors included (4-OCH3bn)^2^m^7^GpppG (27) and (4-Clbn)^2^m^7^GpppG (26), (IC_50_ of 0.89 and 0.98 µM, respectively), and the weakest inhibitors were determined as (4-(diOCH_3_-bn)-tz)^2^m^7^GpppG (28) and (4-CH_3_-th)^2^m^7^GpppG (30), however, a 6.9 and 4.8-fold higher inhibitory potential was still observed relative to the standard. All dinucleotides were used as substrates in an in vitro transcription reaction. Analysis of the incorporation efficiency was evaluated densitometrically after the separation of each sample by UREA-PAGE. The performed tests revealed that all analogues were efficiently incorporated into the RNA transcripts with yield ranging from 88.5 to 67.5% (best for isoxazole-modified dinucleotide, weakest for N2 triazole modification). In the next step, the correctness of the orientation of the analogue’s incorporation into the transcript was investigated. Since RNA polymerases (SP6 and T7) could initiate the transcription reaction in the presence of m^7^GpppG attacking both the 3′-OH group of guanosine and m^7^G, a mixture of transcripts containing m^7^GpppG-RNA and Gpppm^7^G-RNA was generated (Fig. 5) (Pasquinelli et al. 1995). The latter product was translationally inactive and significantly reduced the amount of heterologous protein produced from the RNA preparation. To determine what this looks like when N2-modified analogues are used, an assay was performed with the enzyme hNudt16 (Chrabąszczewska et al. 2021; Grzela et al. 2018). The hydrolytic activity of this enzyme depends on the lack of methylation at the 5′ end of the mRNA, making it possible to distinguish both types of transcripts (e.g. with properly and reversibly incorporated cap structures). The results showed that the level of hydrolysis of (4-(diOCH_3_-bn)-tz)^2^m^7^GpppG-capped RNA (%7.6 ± 1.7) was comparable to that obtained for m_2_^7,3′O^GpppG (8.7 ± 2.0). For the other analogues, the amount of hydrolyzed product ranged from 21.2 ± 5.8 to 33.3 ± 7.2%. Hence, N2-modified analogues not only integrated efficiently in the mRNA transcript but also mostly into the correct orientation. Next, the thermostabilization of eIF4E protein in the presence of the obtained analogues was analyzed using the DSF method. The melting point of dinucleotide-eIF4E complexes were in the temperature range of 49.5‒52.95 °C, and in the case of m^7^GpppG and m_2_^7.3′O^GpppG were 48.1 and 47.53 ºC, respectively. This showed that the new analogues stabilized the protein better than the compounds unmodified at the N2 position. Finally, the efficiency of the translation process was determined using mRNA transcripts carrying the prepared cap analogues. Translation reactions were carried out in the RRL cell-free system and a cellular environment using HEK293 lines. The mRNA transcripts terminated with m^7^GpppG were used as a reference (the translation efficiency was 1.0). The presented results showed that the analogues greatly affected the synthesis of new proteins in both RRL and HEK293 systems (Table 2). The best result was found for the transcript terminated with (4-Cl-bn)^2^m^7^GpppG (26) (3.52 in RRL and 2.29 in HEK293), while that possessing a thiazole ring (30) was the least effective (2.00 in RRL and 1.42 in HEK293). Other analogues in RRL systems gave yields in the range of 3.51‒2.80 in RRL and 2.18‒1.64 in HEK293.

## Conclusion

In conclusion, N2-modified cap analogues consisting of mononucleotides or dinucleotides show favorable biological properties. They exhibit significantly better (in the case of dinucleotides) or similar (in the case of nucleoside monophosphates) abilities to inhibit the translation process in RRL compared to the standard m^7^GpppG. The studies presented show that the modified dinucleotides are effectively incorporated into the structure of the mRNA transcript, and the correctness of their incorporation orientation during this process, allows them to compete with the frequently used ARCA-type analogues. In addition, mRNA transcripts containing cap structures modified within the exocyclic amino group were more efficiently used to synthesize new proteins compared to standard transcripts containing m^7^GpppG or ARCA. Therefore, cap analogues modified at the N2 position can be used in the near future as therapeutics to combat various manifestations of cancer, and become a desirable tool in RNA engineering.

## Data Availability

Not applicable.
